# Soft Elastomeric Capacitor for Angular Rotation Sensing in Steel Components

**DOI:** 10.3390/s21217017

**Published:** 2021-10-23

**Authors:** Han Liu, Simon Laflamme, Jian Li, Caroline Bennett, William N. Collins, Austin Downey, Paul Ziehl, Hongki Jo

**Affiliations:** 1 Department of Civil, Construction and Environmental Engineering, Iowa State University, Ames, IA 50011, USA; laflamme@iastate.edu; 2 Department of Electrical and Computer Engineering, Iowa State University, Ames, IA 50011, USA; 3 Department of Civil, Environmental and Architectural Engineering, The University of Kansas, Lawrence, KS 66045, USA; jianli@ku.edu (J.L.); crb@ku.edu (C.B.); william.collins@ku.edu (W.N.C.); 4 Department of Mechanical Engineering, University of South Carolina, Columbia, SC 29208, USA; austindowney@sc.edu (A.D.); ziehl@cec.sc.edu (P.Z.); 5 Department of Civil and Environmental Engineering, University of South Carolina, Columbia, SC 29208, USA; 6 Department of Civil, Architectural Engineering and Mechanics, The University of Arizona, Tucson, AZ 85721, USA; hjo@arizona.edu

**Keywords:** flexible strain gauge, angular rotation, angular motion sensing, strain monitoring, capacitor, bending strain, complex geometry, out-of-plane deformation

## Abstract

The authors have previously proposed corrugated soft elastomeric capacitors (cSEC) to create ultra compliant scalable strain gauges. The cSEC technology has been successfully demonstrated in engineering and biomechanical applications for in-plane strain measurements. This study extends work on the cSEC to evaluate its performance at measuring angular rotation when installed folded at the junction of two plates. The objective is to characterize the sensor’s electromechanical behavior anticipating applications to the monitoring of welded connections in steel components. To do so, an electromechanical model that maps the cSEC signal to bending strain induced by angular rotation is derived and adjusted using a validated finite element model. Given the difficulty in mapping strain measurements to rotation, an algorithm termed angular rotation index (ARI) is formulated to link measurements to angular rotation directly. Experimental work is conducted on a hollow structural section (HSS) steel specimen equipped with cSECs subjected to compression to generate angular rotations at the corners within the cross-section. Results confirm that the cSEC is capable of tracking angular rotation-induced bending strain linearly, however with accuracy levels significantly lower than found over flat configurations. Nevertheless, measurements were mapped to angular rotations using the ARI, and it was found that the ARI mapped linearly to the angle of rotation, with an accuracy of 0.416${}^{\circ }$.

## 1. Introduction

The rapid growth of the electronics industry and advanced materials has enabled the development of flexible electronics, empowering new measurement capabilities over complex and highly deformable surfaces. Of interest to this paper are thin-film based flexible devices capable of mechanical strain measurement, termed flexible strain sensors [1,2]. These devices can function on different sensing principles, with mechanisms based on capacitance [3,4,5], resistance [6,7], piezoelectrics/triboelectrics [8,9], and transistors [10,11], and with example applications in the areas of biomechanical engineering [12,13], wearable sensors [14,15], and structural health monitoring [16,17]. Flexible sensors can be fabricated using conductive polymers [18,19] and silicone [20,21], sometimes doping the matrix with nanoparticles [22,23,24,25], to improve on electrical properties.

A key advantage of flexible sensors is their capability to sustain large deformations, making them ideal candidates for applications to irregular or complex geometries. They can advantageously be applied over corners, welds, and curved and rugged surfaces. Here, we study the use of a flexible sensor for the monitoring of angular motion in steel components. Other angular motion sensing technologies have been studied in the literature. This includes a tube-based triboelectric–electromagnetic sensor that integrates magnets and a coil with flexible foam [26], a planar microwave sensor built from a split-ring resonator and a microstrip line [27], an ultralight, flexible, and super compressible sensor fabricated using reduced graphene oxide-based lamellar carbon aerogels [28], graphene platelets (GnPs) and multi-walled carbon nanotubes (MWCNTs)-based films [29], a highly stretchable sensor fabricated by depositing carboxylic MWCNTs onto bacterial cellulose substrate [30], and a flexible strain sensor fabricated by incorporating platinum (Pt) constituent material into polyimide films [31].

The authors have previously proposed a soft elastomeric capacitor (SEC) that transduces strain into a measurable change in capacitance. Key advantages of the SEC are its high compliance, with a demonstrated linear strain sensing range up to 20% [32], and its high scalability due to its low cost and ease of fabrication. The SEC is a parallel plate capacitor constituted by layering styrene–ethylene–butylene–styrene (SEBS)-based thin-films filled with carbon black (CB) to form the conductive plate layers, and with filled with titania to form the dielectric layer. The technology has been applied to many structural health monitoring applications, in particular for the monitoring of fatigue cracks in steel [33,34]. Recently, a new generation SEC, termed corrugated soft elastomeric capacitor (cSEC), was engineered by texturing the top surface of the dielectric in order to tune the in-plane stiffness and thus the transverse Poisson’s ratio. Laboratory investigations have shown that adding such corrugation significantly improved the sensing performance of the SEC [35]. In particular, tests results reported a gauge factor of 1.61, minimum strain resolution of ±14.1 με, and minimum detectable fatigue crack size of 0.31 mm on compact tension specimens [36].

In this paper, we leverage the high compliance of the cSEC to measure angular motion in steel components by deploying the sensor in a folded configuration. While the long-term goal is to use the sensor to detect and quantify cracks in corner welds, the objective of this paper is to characterize the cSEC signal when used for angular motion sensing. The study is performed on a hollow structural section (HSS) subjected to axial strain causing angular motions at the corners of the cross-section. An electromechanical model is developed and validated against experimental results. Then, an index termed angular rotation index (ARI) is proposed to fuse measurements into a metric quantifying the level of angular rotation.

The rest of the paper is organized as follows. Section 2 provides the background on cSEC technology and typical types of bending strains leveraged on thin panels. Section 3 presents the methodology, including a description of the experimental procedure, the numerical model, the derivation of cSEC’s electromechanical model under bending strain, and the proposed algorithm for quantifying angle of rotations. Section 4 presents and discusses results, starting with the validation of the numerical model, followed by an analysis of the signal study from the experimental investigation, and by the discussion of results obtained from the algorithm. Section 5 concludes the paper.

## 2. Background

This section provides the necessary background on the cSEC technology along with the derivation of an extended electromechanical model adapted for angular motion sensing and discusses a four-step algorithm used in assessing and quantifying the angle of rotations.

### 2.1. cSEC Technology

**Figure 1:**
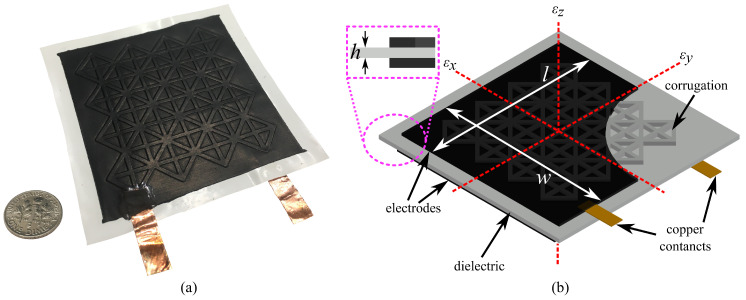
(**a**) Picture of a 76 mm × 76 mm cSEC with a reinforced pattern; (**b**) schematic of a cSEC of thickness *h* and a section of the electrode layer with electrode area l×w (black layer).

A cSEC fabricated with a reinforced corrugation pattern is presented in Figure 1a. Its sensing principle is based on a measurable change in capacitance provoked by strain-induced deformations in the sensor’s geometry. The fabrication process is described in detail in [35]. Briefly, the dielectric of the sensor is constituted by a matrix of styrene–ethylene–butylene–styrene (SEBS) filled with titania dioxide, and the conductive plates by SEBS filled with carbon black. The dielectric is drop-cast in a steel mold to form the corrugation, and the conductive layers are painted onto each side of the dried dielectric. Two copper tapes are adhered onto the painted electrodes to create electrical connections, and a thin layer of PELCO conductive glue (No: 16050, TED Pella, INC., Redding, CA, USA) is applied over the copper tapes to ameliorate the electrical contact. In what follows, an extended electromechanical model suitable for measuring bending deformations and the ARI algorithm used to relate measurements to angular motions are presented.

### 2.2. Electromechanical Model

In prior work, the cSEC was utilized to measure strain associated with in-plane deformation in the sensor, for instance, from pure axial elongations or widening of cracks. The electromechanical model of the sensor for in-plane deformation is derived as follows. Assuming under a low measurement frequency (<1 kHz), the cSEC can be modeled as a non-lossy parallel plate capacitor of initial capacitance $C_{0}$

(1) $$C_{0}=e_{0}e_{r}\frac{A}{h}$$

where $e_{0}=8.854$ pF/m is the vacuum permittivity, $e_{r}$ is the relative permittivity, *h* is the thickness of the dielectric, and *A* is the electrode area of length *l* and width *d*, as annotated in Figure 1b.

**Figure 2:**
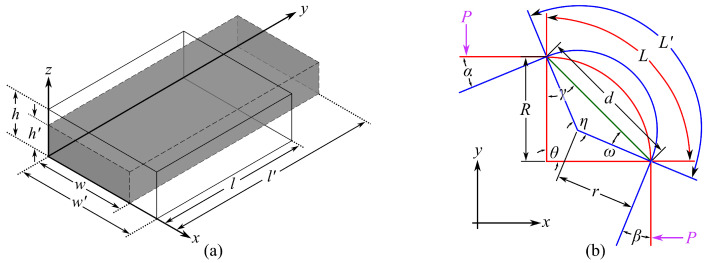
(**a**) Small cuboid unit of the mesh element; (**b**) deformation of an arc under compression.

To derive the electromechanical model applied to angular motions, consider a small section of a cSEC as illustrated in Figure 2a. The section is of initial length *l*, width *w*, and thickness *h*, and the strain is assumed to be distributed uniformly along the section. An incremental stretch along the *y* direction produces a longer length $l^{\prime }$, smaller width $w^{\prime }$, and smaller thickness $h^{\prime }$, where the prime denotes a deformed dimension. Here, the capacitance response, $\Delta C/C_{0}$ can be expressed as:

(2) $$\frac{\Delta C}{C_{0}}=\frac{C_{1}-C_{0}}{C_{0}}=\frac{e_{0}e_{r}\left(\frac{A^{\prime }}{h^{\prime }}-\frac{A}{h}\right)}{e_{0}e_{r}\frac{A}{h}}=\frac{A^{\prime }h-Ah^{\prime }}{Ah^{\prime }}$$

where $C_{1}$ is the capacitance and $A^{\prime }$ is the deformed sensing area of the deformed section. Substituting A=lw and $A^{\prime }=l^{\prime }w^{\prime }$ into Equation (2), one obtains:

(3) $$\frac{\Delta C}{C_{0}}=\frac{l^{\prime }w^{\prime }h-lwh^{\prime }}{lwh^{\prime }}$$

Assuming small changes in geometry, differentiating Equation (1) with respect to length *l* and width *w* yields an expression for the relative change in capacitance $\Delta C/C_{0}$ with the in-plane strains $\varepsilon_{x}$ and $\varepsilon_{y}$, and out-of-plane strain $\varepsilon_{z}$:

(4) $$\frac{\Delta C}{C_{0}}=\left(\frac{\Delta l}{l}+\frac{\Delta w}{w}-\frac{\Delta h}{h}\right)=\left(\frac{l^{\prime }-l}{l}+\frac{w-w^{\prime }}{w}-\frac{h-h^{\prime }}{h}\right)=\varepsilon_{y}+\varepsilon_{x}-\varepsilon_{z}$$

where the subscript denotes the direction of strain ε along the principal axes, as indicated in Figure 1b. Using the expressions $l^{\prime }=\left(1+\varepsilon_{x}\right)l$, $w^{\prime }=\left(1+\varepsilon_{y}\right)w$, and $h^{\prime }=\left(1+\varepsilon_{z}\right)h$, Equation (3) becomes:

(5) $$\frac{\Delta C}{C_{0}}=\frac{\left(1+\varepsilon_{x}\right)\left(1+\varepsilon_{y}\right)}{1+\varepsilon_{z}}-1$$

Using Hooke’s Law under plane stress assumption with ν denoting the Poisson’s ratio of the SEBS, the stress along the *z*-axis can be written as:

(6) $$\varepsilon_{z}=-\frac{\nu }{E}\left(\sigma_{x}+\sigma_{y}\right)=-\frac{\nu }{1-\nu }\left(\varepsilon_{x}+\varepsilon_{y}\right)=\frac{1}{\left(1+\varepsilon_{x}\right)\left(1+\varepsilon_{y}\right)}-1$$

substituting Equation (6) into Equation (5) gives:

(7) $$\frac{\Delta C}{C_{0}}={\left(1+\varepsilon_{x}\right)}^{2}{\left(1+\varepsilon_{y}\right)}^{2}-1$$

The use of a corrugated pattern introduces an orthotropic material behavior. Here, the transverse Poisson’s ratio $\nu_{xy}$ of a cSEC in a free-standing configuration is taken as:

(8) $$\nu_{xy}=-\frac{\varepsilon_{y}}{\varepsilon_{x}}$$

when the sensor is fully adhered onto a monitored material, the transverse Poisson’s ratio $\nu_{xy}$ will be influenced under a composite effect, particularly, the stiffness of the monitored material and the level of adhesion. Therefore, the transverse Poisson’s ratio under composite effect $\nu_{xy,c}$ can be expressed as a function of weighted Poisson’s ratio and written as:

(9) $$\nu_{xy,c}=-\frac{a\nu_{xy}+b\nu_{m}}{a+b}=-\frac{\varepsilon_{y,c}}{\varepsilon_{x,c}}$$

where $\nu_{m}$ is the Poisson’s ratio of monitored material, 0 ≤ *a* ≤ 1 and 0 ≤ *b* ≤ 1 are weights such that *a* + *b* = 1, and $\varepsilon_{x,c}$ and $\varepsilon_{y,c}$ are the in-plane strains under composite effect. For the free-standing cSEC, *a* = 1 and *b* = 0, and for the material with high stiffness (e.g., steel or concrete), *a*≈ 1 and *b*≈ 0. Substituting Equation (9) into Equation (7) yields:

(10) $$\frac{\Delta C}{C_{0}}={\left(1+\varepsilon_{x,c}\right)}^{2}{\left(1-\nu_{xy,c}\;\cdot \;\varepsilon_{x,c}\right)}^{2}-1$$

Assuming that the majority of strain deformation ($\varepsilon_{x,c}$) in the sensor is attributed to the rotation of the arc-length, and Equation (10) can be further refined by evolving with the angular rotation. Figure 2b is the diagram of an arc of initial arc angle θ and chord length *d*. In the experimental section, values of $\theta =89.85^{\circ }$ and $\theta =90.05^{\circ }$, and d=10.2 mm and d=10.1 mm were obtained for the left and right corners, respectively. The initial arc length *L* can be written:

(11) $$L=\theta \;\cdot \;R=\theta \;\cdot \;\frac{d\;\cdot \;\sin(\gamma )}{\sin(\theta )}=\theta \;\cdot \;\frac{d\;\cdot \;\sqrt{\frac{1-\cos(180^{\circ }-\theta )}{2}}}{\sin(\theta )}$$

where *R* is the radius of curvature and $\gamma =(180^{\circ }-\theta )/2$.

Consider two concentrated loads *P* acting at the free ends of the arc, and the deformed arc central angle and radius be η and *r*, as illustrated in Figure 2b. Angle η can be expressed as a function of the angular rotation α and β (also known as angle of rotation, angle of inclination, and angle of slope) written as:

(12) η=θ+α+β

where α and β are the angle of rotations between the *x* and *y* axes and the tangent at the tip of the deflected arcs in *x* and *y* directions, respectively. The deformed arc length $L^{\prime }$ can be expressed as:

(13) $$L^{\prime }=\eta \;\cdot \;r=\left(\theta +\alpha +\beta \right)\;\cdot \;\frac{d\;\cdot \;\sin(\omega )}{\sin(\eta )}=\left(\theta +\alpha +\beta \right)\;\cdot \;\frac{d\;\cdot \;\sqrt{\frac{1-\cos(180^{\circ }-\theta -\alpha -\beta )}{2}}}{\sin(\theta +\alpha +\beta )}$$

with

(14) $$\omega =\frac{180-\eta }{2}=\frac{180^{\circ }-\theta -\alpha -\beta }{2}$$

It follows that $\varepsilon_{x,c}$ in Equation (10) can be taken as bending strain $\varepsilon_{x}$ and written as the function of *L* and $L^{\prime }$ derived in Equations (11) and (13):

(15) $$\begin{array}{cc} \varepsilon_{x,c} & =\varepsilon_{x}=\frac{\Delta L}{L}=\left(L^{\prime }-L\right)/L\\ \; & =\frac{\left(\left(\theta +\alpha +\beta \right)\;\cdot \;\frac{d\;\cdot \;\sqrt{\frac{1-\cos(180^{\circ }-\theta )}{2}}}{\sin(\theta +\alpha +\beta )}-\theta \;\cdot \;\frac{d\;\cdot \;\sqrt{\frac{1-\cos(180^{\circ }-\theta -\alpha -\beta )}{2}}}{\sin(\theta )}\right)}{\theta \;\cdot \;\frac{d\;\cdot \;\sqrt{\frac{1-\cos(180^{\circ }-\theta )}{2}}}{\sin(\theta )}} \end{array}$$

### 2.3. ARI Algorithm

**Figure 3:**
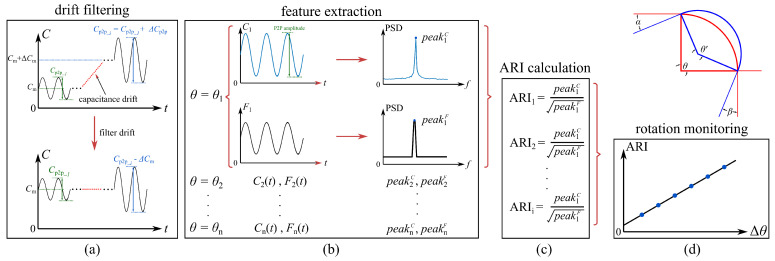
Four-step algorithm used on monitoring angle of rotation: (**a**) data acquisition and drift filtering; (**b**) feature extraction; (**c**) construction of ARI; (**d**) rotation monitoring.

The angular rotation index (ARI) algorithm is developed to fuse cSEC data into a scalar relating to the angle of rotation. The algorithm includes four consecutive steps, illustrated in Figure 3 and discussed in what follows.

The first step consists of filtering drifts out of the measurements, a common issue found in strain gauges used over long periods of time (Figure 3a). To do so, the change in mean capacitance $\Delta C_{m}$ is computed and subtracted from each measurement segment to align signals with the initial mean capacitance $C_{m}$. In field applications, the cSEC may be affected by variations in temperature and humidity. These environmental effects can be filtered out, for example, through the design of a Wheatstone bridge configuration [37].

The second step consists of extracting features (Figure 3b). These features correspond to the peak amplitudes of capacitance (peak${}_{i}^{C}$) and force (peak${}_{i}^{F}$) from the ith measurement taken in the power spectral density (PSD) as the frequency domain signal is less sensitive to the noise content of the measurements, and used to represent the peak-to-peak (P2P) amplitudes in the time domain. The utilization of these features is useful for filtering out signal drifts (e.g., temperature effects) and shifts (e.g., from a loose cable).

The third step consists of fusing features into the ARI (Figure 3c). Because the load range directly affects the P2P $\Delta C/C_{0}$ of the cSEC, peak${}_{i}^{C}$ is normalized by taking the ratio to the square root of the peak force $\sqrt{{\mathrm{peak}}_{i}^{F}}$ to make the ARI input-independent, with the ARI of the *i*th measurement segment being ${\mathrm{ARI}}_{i}={\mathrm{peak}}_{i}^{C}/\sqrt{{\mathrm{peak}}_{i}^{F}}$. The square root is taken in this equation to reduce heteroscedasticity of the residuals in linear regression and weaken the effect of the non-linear relationship in Equation (15). Mathematically, the ARI represents the level of the angular rotation induced by the P2P amplitude under a unit excitation load.

The fourth step consists of correlating the ARI with the angle of rotation (Figure 3d). This can be done by characterizing the relationship between ARI and Δθ, therefore enabling the identification of Δθ online in real-time.

## 3. Methodology

This section presents the general methodology applied in this research. First, the experimental setup along with the procedure used to study the cSEC’s signals are described. Second, the finite element model (FEM) is presented, including the geometries, material properties, and boundary conditions. Third, the algorithm used in generating synthetic capacitance data is discussed.

### 3.1. Experimental Test

**Figure 4:**
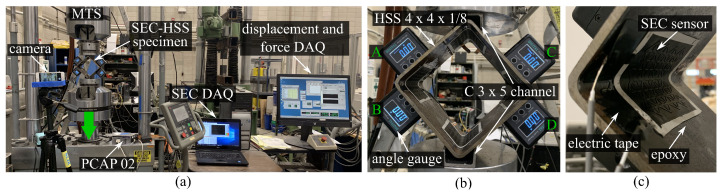
(**a**) Overall experimental configuration (green arrow indicates the loading direction); (**b**) zoom on the front side of HSS specimen; (**c**) close-up view of the inner surface, right corner.

**Figure 5:**
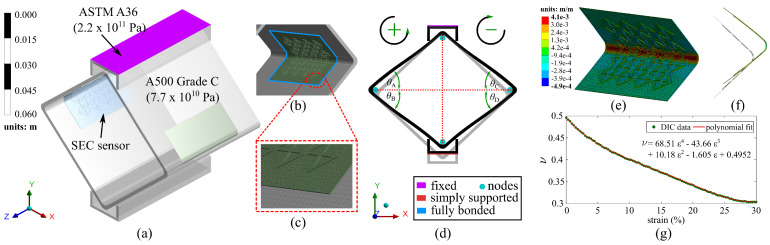
(**a**) Schematic of the numerical model showing the general geometry and of the HSS specimen; (**b**) boundary conditions of the cSEC; (**c**) mesh type and mesh distribution; (**d**) simulated deformation and boundary conditions of the HSS specimen; (**e**) normal elastic distribution of the cSEC within a folded configuration; (**f**) front section view of the sensor showing the deformation under angular motion; (**g**) strain-dependent nonlinear transverse Poisson’s ratio of SEBS.

The experimental study focused on characterizing the capacitance response of the cSEC in a folded configuration using a 101.6 mm × 101.6 mm × 3.175 mm hollow structural section (HSS) specimen (A500 Grade C). The HSS section is used to mimic the curved surface of an orthogonal joint in a connection. Figure 4a shows the overall experimental setup, and Figure 4b is a close-up view of the front side of the HSS specimen. The inner surface of the HSS specimen was sanded using 1000 grit sandpaper and cleaned with acetone. After, as shown in Figure 4c, four cSECs were glued in folded configurations by adhering the flat surface onto the inner surface of the curved corners using an off-the-shelf bi-component epoxy (JB Weld) so that the sensor was in full contact with the arc surface, which allowed the measurement of angular motion. Wires were fixed with electrical tape to be electrically insulated. Two C 3 × 5 steel channels (Gr. A36 steel) were placed over the top and bottom corners to affix the HSS to a closed-loop servo-hydraulic testing machine (MTS model 312.41 with a TestStar IIm controller) equipped with model 647 HydraulicWedge Grips. Four digital angle gauges, labeled with A, B, C, and D in Figure 4b, with a measurement resolution of 0.05${}^{\circ }$ and a minimum reaction time of 0.1 s were installed above and below the left and right corners to measure localized angular rotations. The measurements from each angle gauge were assigned to be negative for clockwise rotations and positive for counterclockwise rotations, as indicated in Figure 5d.

A preload of 0.05 kN was applied on the HSS specimen prior to each test to obtain a compression–compression mode, and the specimen was subjected to a displacement-controlled harmonic excitation at a constant frequency of 1 Hz. Ten tests were conducted, each lasting 120 cycles, but at different displacement amplitudes: 0.5, 1, 1.5, 2, 2.5, 3, 3.5, 4, 4.5, and 5 mm. A digital camera was placed in front of the specimen to simultaneously record the angular rotations measured by the angle gauges during testing, and the frame rate was set as 30 fps. Load and displacement data were recorded from the MTS at 20 samples/second, and cSEC capacitance data was sampled at 80 samples/second using an off-the-shelf data acquisition board (ACAM PCAP02).

### 3.2. Numerical Model

To numerically characterize the sensor’s response under angular motion-induced bending strain, a 3D nonlinear FEM of the experimental setup was constructed in ANSYS 2020 R2 [38]. As shown in Figure 5a, the HSS specimen and the C-shape channels were respectively assigned as ASTM A500 Grade C steel and A36 steel. The material properties used in the FEM simulations are listed in Table 1. The cSEC was modeled as a corrugated film with a substrate layer of 0.3 mm and corrugated height of 0.35 mm, and the SEBS material was defined as an isotropic polymer with a Young’s Modulus of *E* = 0.41 MPa and with the strain-dependent nonlinear Poisson’s ratio ν experimentally obtained in [35] using digital image correlation (DIC) tests and plotted in Figure 5g.

As illustrated in Figure 5c, the sensor and HSS specimens were meshed by using the tetrahedrons and multizone mesh method, respectively. The mesh size was set as 0.2 mm following the results of a convergence study, resulting in a total of 7013 mesh elements on the sensor. Boundary conditions of the cSEC-HSS specimen are presented in Figure 5b,d. The cSEC was modeled as fully bonded on the inner surface of the HSS specimen to simulate a full adhesion, and the HSS specimen was constrained by a fixed support (restraining *x*, *y*, and *z* translational degrees-of-freedom) at the top C-shape channel and simply supported (restraining *x* and *y* translational degrees-of-freedom) at the bottom of the C-shape channel. Both C-shape channels were assigned as frictional contacts at the HSS interface, and the frictional coefficient was defined as 0.78 obtained from literature [39]. Four pairs of nodes were set at each corner of the HSS to generate synthetic measurements of localized angular rotations ($\Delta \theta_{i}$), corresponding to the measured states from the four digital angle gauges used in the experimental tests. A 1 Hz harmonic excitation with constant displacement levels was applied on the base surface of the bottom C-shape channel section to simulate the loading protocol used in the laboratory environment.

### 3.3. Simulation of cSEC Measurements

**Figure 6:**
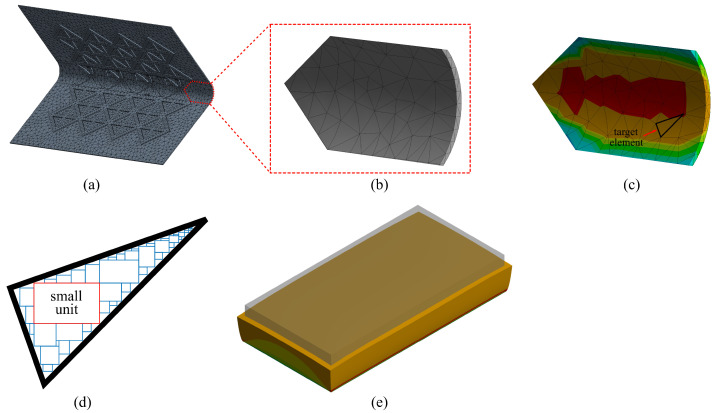
Schematic drawings show: (**a**) meshed cSEC; (**b**) details of partitioned elements; (**c**) normal elastic strain distributed on target element; (**d**) selected mesh element; (**e**) small cuboid unit of the mesh element under deformation.

Figure 6a,b present the distribution of the generated mesh elements on cSEC. Equation (2) indicates the capacitance response of the sensor can be directly obtained from the change in its sensing area. This principle is used to generate synthetic measurements in FEM simulations. To accomplish this, a sensor is meshed into *P* elements, and each element is sub-divided into *Q* cuboids (Figure 6d), where each cuboid is considered small enough such that the strain field can be assumed as uniform within the cuboid. The relative change in capacitance of a given cuboid (Figure 6e) is taken as:

(16) $$\begin{array}{cc} \frac{\Delta C^{p,q}}{C_{0}^{p,q}} & ={\left(1+\varepsilon_{x,c}^{p,q}\right)}^{2}{\left(1-\nu_{xy,c}^{p,q}\;\cdot \;\varepsilon_{x,c}^{p,q}\right)}^{2}-1\\ \; & =\frac{A^{p,q}}{A_{0}^{p,q}} \end{array}$$

where superscript p,q indicates quantities associated with the *q*th cuboid of the *p*th mesh element. The capacitance response of the meshed region (Figure 6c) is obtained through the summation of the capacitance of every cuboid over every mesh element:

(17) $$\frac{\Delta C}{C_{0}}=\frac{1}{p\;\cdot \;q}\sum_{p=1}^{P}\sum_{q=1}^{Q}\frac{\Delta A^{p,q}}{A_{0}^{p,q}}=\frac{1}{p\;\cdot \;q}\sum_{p=1}^{P}\sum_{q=1}^{Q}\frac{\Delta C^{p,q}}{C_{0}^{p,q}}$$

Figure 5e presents the normal elastic strain distribution numerically obtained from the FEM under a 4 mm displacement. Applying Equation (16) shows that up to 93.6% of the total elastic strain under the angular motion is distributed around the sensing area in contact with the corner of the HSS specimen (as shown in Figure 5e). Therefore, it verified the hypothesis in Equation (11) that the electromechanical model of the cSEC under the angular bending deformation can be derived by accounting only for strain occurring along the arc length of the corner.

## 4. Results and Discussion

This section validates the numerical model using experimental data and analyses experimental results demonstrating the capability of the cSEC for monitoring both the rotation-induced bending strain and angular rotations.

### 4.1. Numerical Model Validation

**Figure 7:**
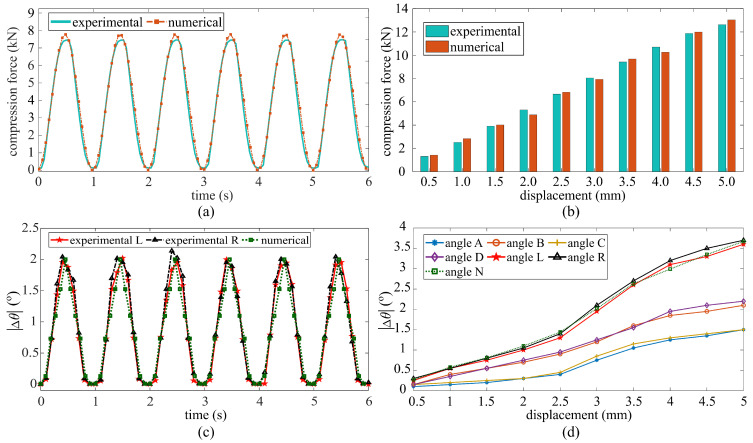
Comparison of the compression forces obtained numerically and experimentally: (**a**) time series under a 3.0 mm maximum displacement amplitude, and (**b**) average maximum compression force under each maximum displacement amplitude; (**c**) comparison of angular rotation ($\Delta \theta_{i}$) under a 3.0 mm maximum displacement amplitude; (**d**) absolute values of angular rotations ($\Delta \theta_{i}$) as a function of applied displacement.

The validation of the numerical model was conducted by comparing experimental to numerical results using the applied compression forces and measured angle of rotation Δθ. Figure 7a is a time series plot comparing results under a displacement of 3.0 mm maximum amplitude, and Figure 7b summarizes results under each maximum displacement level, with experimental results shown as the averaged maximum compression force over 120 cycles. There is a close match between experimental tests and the FEM, with a fitted root mean square error (RMSE) and mean absolute percentage error (MAPE) of 4.03% and 5.29% in Figure 7a,b, respectively. Slight discrepancies are attributable to the sliding of the C-shape connecting the HSS to the MTS.

Figure 7c compares results for the angle of rotation in time series under a maximum displacement amplitude of 3.0 mm, where L and R denote the left and right angles, respectively. Figure 7d plots the measured Δθ under each maximum displacement amplitude, with results presented for each angle gauge. It can be noticed that the angular rotations measured by gauges B ($\Delta \theta_{B}$) and D ($\Delta \theta_{D}$) are consistently higher than those measured by gauges A ($\Delta \theta_{A}$) and C ($\Delta \theta_{C}$), attributable to the asymmetric boundary conditions. The left angle ($\Delta \theta_{L}$) and right angle ($\Delta \theta_{R}$) were calculated as the summation of the absolute value of angles A, C, ($\left|\Delta \theta_{A}\right|$+$\left|\Delta \theta_{C}\right|$) and angles B, D, ($\left|\Delta \theta_{B}\right|$+$\left|\Delta \theta_{D}\right|$) respectively, and angle N ($\Delta \theta_{N}$) represents the results obtained from the numerical model. There is good agreement between experimental and numerical results, with the RMSE values of 5.33% (left angle) and 6.41% (right angle) in Figure 7c and a maximum difference of 7.29% in Figure 7d under the displacement of 4.5 mm. Overall, results show that experimental results can be adequately reproduced numerically.

### 4.2. Bending Strain Monitoring

**Figure 8:**
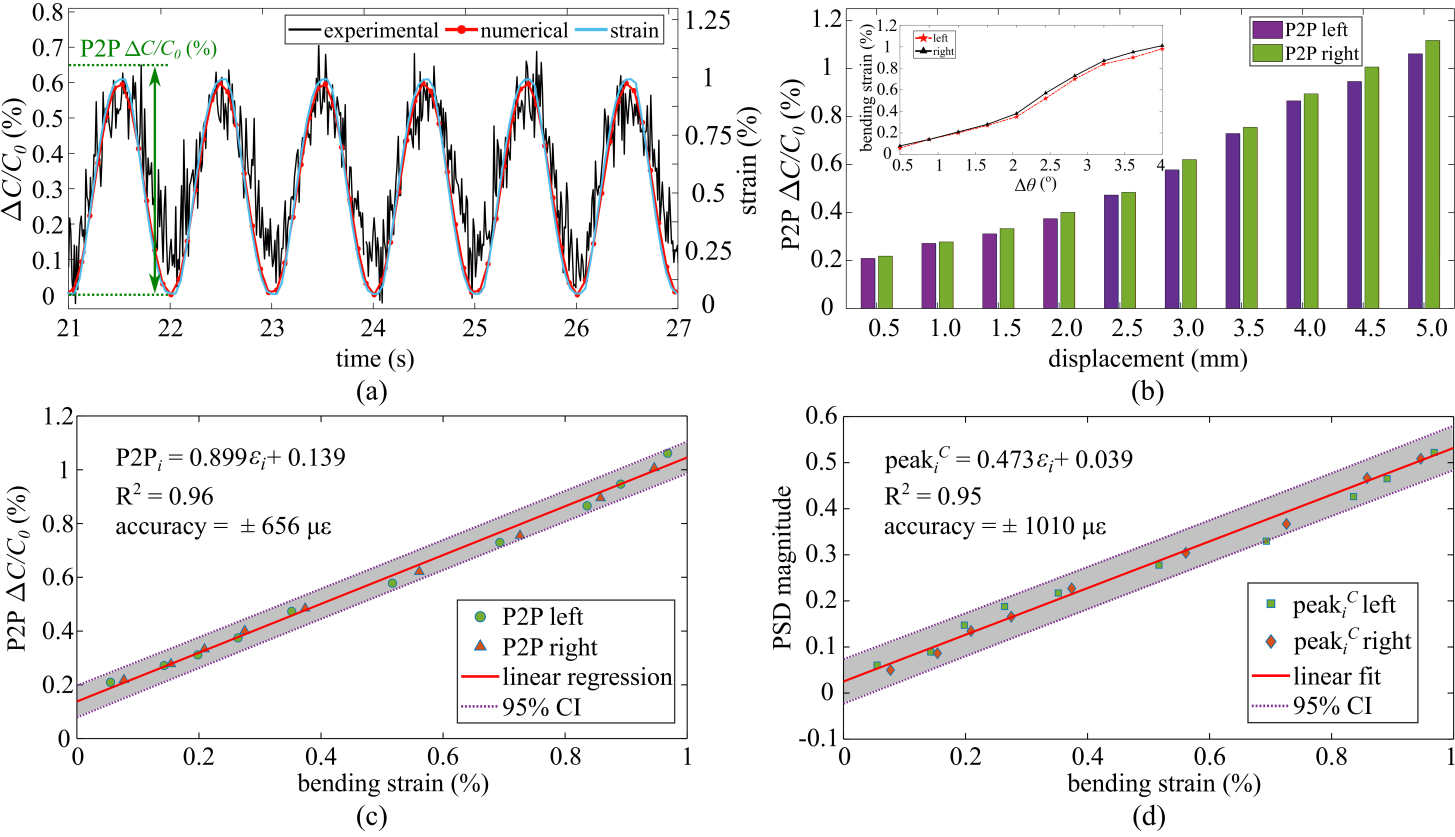
(**a**) Comparison of experimental and numerical signals for $\Delta C/C_{0}$ under a 4.0 mm maximum displacement; (**b**) P2P $\Delta C/C_{0}$ amplitude as a function of displacement with the inset showing bending strain as the function of angular rotations (Δθ); (**c**) linear regression of the P2P amplitudes with respect to bending strain; (**d**) linear regression of the PSD amplitudes with respect to bending strain.

Figure 8a is a typical time series plot of the raw data measured from the cSEC (installed at the right angle) compared against the numerical response under the maximum displacement of 4 mm. The first 20 s were discarded to eliminate the early-stage noise in the signal. Quantities $\Delta \theta_{C}$ and $\Delta \theta_{D}$ were substituted into Equation (15) as α and β to convert the measured angular rotation $\Delta \theta_{R}$ into bending strain $\varepsilon_{x,c}$, represented by the blue line in Figure 8a for which a linear interpolation was used to create a smooth curve. Strain obtained from the numerical capacitance response was also converted to bending strain ($\varepsilon_{x,c}$) and represented by a red-circle line in the Figure. There is a good fit between the experimental and numerical capacitance responses, with an RMSE value of 4.98%, showing that the electromechanical model can be used to estimate bending strain.

Figure 8b is a bar chart comparing the averaged 120 cycles peak-to-peak (P2P) relative capacitance $\Delta C/C_{0}$ amplitudes (P2P illustrated in Figure 8a) under each maximum displacement, where a higher displacement correlates with a larger angular deformation and thus larger bending strain. It was found that the magnitudes of the P2P $\Delta C/C_{0}$ increase with increasing maximum displacement. The inset in Figure 8b shows bending strain versus angular rotations (Δθ), where bending strains were also calculated by using Equation (15) with reported $\Delta \theta_{i}$ in Figure 7b. Figure 8c,d plot the P2P $\Delta C/C_{0}$ and peak amplitudes of the PSD of capacitance (peak${}_{i}^{C}$) as a function of bending strain, respectively. Fitted linear regressions have R${}^{2}$ values of 96.1% (P2P $\Delta C/C_{0}$) and 94.9% (peak${}_{i}^{C}$), and the 95% confidence interval (CI) bounds result in an accuracy of ± 656 με (P2P) and ± 1010 με (PSD), respectively. This is significantly more than the levels reported in prior work on the cSEC under 54 με [36] for measuring bending-induced and crack-induced strain over a flat surface. This could be explained by the change in local thickness of the cSEC, additional strain induced in the system that is ignored by the model, and the imperfect adhesion of the sensor during the hand-application process.

Yet, it is evident that cSEC signal can be used to quantify the angular rotation-induced bending strain through a linear relationship. However, mapping the cSEC measurements to rotations is more difficult, as observed through Equation (15) and the nonlinear relationship plotted in the inset of Figure 8b. The ARI algorithm, developed for that purpose, is verified in the upcoming subsection.

### 4.3. Angular Rotation Monitoring

**Figure 9:**
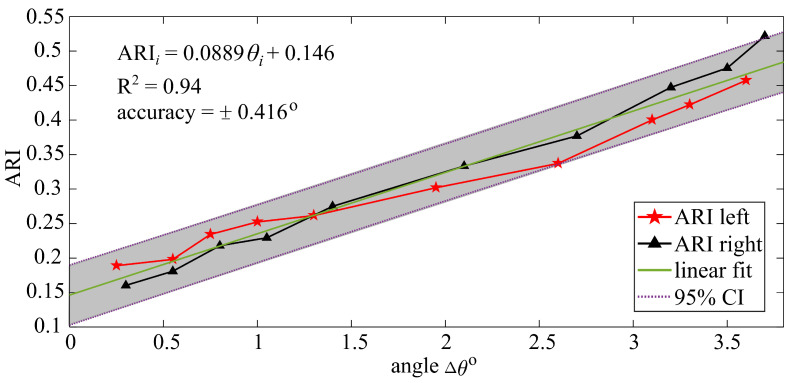
Linear regression of ARI with respect to angular rotation $\Delta \theta_{i}$.

Figure 9 plots the computed ARI as a function of angle of rotations (Δθ) for the left and right sensors. A desired linear relationship between the ARI and Δθ was found on both sets of measurements (left and right sensors), which verifies that the ARI could be used as a metric to quantify angular rotation. The 95% CI bound in terms of ARI and angular rotations $\Delta \theta_{i}$ yields an accuracy of $\pm 0.416^{\circ }$, which compares well with off-the-shelf tiltmeters. Overall, the sensor let to large strain readings in the folded configuration with a poor resolution, nevertheless results from the ARI showed good accuracy in terms of degrees. Additional tests on different cross-section geometries would be required to further study the quality of the linear regression. This is left to future work. It should also be remarked that this work only considered rotation-induced bending strain and that the presence of a fatigue crack, for instance, if the sensor was installed over a corner weld, may induce additional kinetics. This is also left to future work.

## 5. Conclusions

This paper studied the use of an ultra compliant flexible strain gauge, termed corrugated soft elastomeric capacitor (cSEC), to measure angular rotations. The objective was to understand the electromechanical behavior of the sensor when installed in a folded configuration, with applications foreseen for fatigue crack monitoring of corner welds and other complex geometries.

Experiments were conducted using an HSS specimen to mimic the curved surface of an orthogonal joint in a connection. Angular deformations at the corners of the HSS section were generated by subjecting the specimen to compression force. cSEC sensors were fully adhered onto the inner corner surface in a folded configuration. A dynamic excitation was applied on the HSS specimen, and the cSEC capacitance response was investigated under several different displacement amplitudes. A finite element model (FEM) was constructed and validated by comparing experimental and numerical data. Then, the elastic strain distributed on the sensor was numerically simulated, and the electromechanical model was developed for rotation-induced bending strain. An algorithm, termed angular rotation index (ARI), was proposed to map cSEC data to rotations. Experimental data were used to evaluate cSEC performance at measuring rotation-induced strain as well as the ARI’s promise at mapping measurements to angular rotations.

Results showed that the peak-to-peak (P2P) relative capacitance amplitude and power spectral density (PSD) peak of the capacitance (peak${}_{i}^{C}$) increased with the increasing angular rotation, and those quantities linearly related with bending strain, however with accuracy levels significantly lower than found over flat configurations. Nevertheless, measurements were mapped to angular rotations using the ARI, and it was found that the ARI mapped linearly to the angle of rotation, with an accuracy of 0.416${}^{\circ }$ that compares well with off-the-shelf tiltmeters.

Overall, results from this investigation demonstrated that the cSEC could be used as an angular rotation sensor. Future work is to include further testing on different specimens and the investigation of the effects of fatigue cracks when applied over a fillet weld.

## Figures and Tables

**Table 1:** Assigned properties for ASTM A500 Grade C and Gr. A36 steel.

Material Property	A500 Grade C	Gr. A36
density	7800 kg/m${}^{3}$	7850 kg/m${}^{3}$
Young’s modulus	$7.72\times 10^{10}$ Pa	$2.02\times 10^{11}$ Pa
Poisson’s ratio	0.31	0.32
bulk modulus	158 GPa	167 GPa
shear modulus	80.2 GPa	76.9 GPa
tensile yield strength	315 MPa	420 MPa
tensile ultimate strength	425 MPa	560 MPa

## Data Availability

The data presented in this study are available on request from the corresponding author.

## Abbreviations

The following abbreviations are used in this manuscript:

SHM	Structural Health Monitoring
SEC	Soft Elastomeric Capacitor
cSEC	Corrugated Soft Elastomeric Capacitor
HSS	Hollow Steel Section
FE	Finite Element
SEBS	Styrene–Ethylene–Butylene–Styrene
LVDT	Linear Variable Displacement Transducer
P2P	Peak-to-Peak
PSD	Power Spectral Density
ARI	Angular Rotation Index

## References

[1] Amjadi M., Kyung K.U., Park I., Sitti M. (2016). Stretchable, Skin-Mountable, and Wearable Strain Sensors and Their Potential Applications: A Review. Adv. Funct. Mater..

[2] Seyedin S., Zhang P., Naebe M., Qin S., Chen J., Wang X., Razal J.M. (2019). Textile strain sensors: A review of the fabrication technologies, performance evaluation and applications. Mater. Horiz..

[3] Atalay O. (2018). Textile-Based, Interdigital, Capacitive, Soft-Strain Sensor for Wearable Applications. Materials.

[4] Xu H., Lv Y., Qiu D., Zhou Y., Zeng H., Chu Y. (2019). An ultra-stretchable, highly sensitive and biocompatible capacitive strain sensor from an ionic nanocomposite for on-skin monitoring. Nanoscale.

[5] Chhetry A., Sharma S., Yoon H., Ko S., Park J.Y. (2020). Enhanced Sensitivity of Capacitive Pressure and Strain Sensor Based on CaCu_3_Ti_4_O_12_ Wrapped Hybrid Sponge for Wearable Applications. Adv. Funct. Mater..

[6] Li W., Xu F., Liu W., Gao Y., Zhang K., Zhang X., Qiu Y. (2018). Flexible strain sensor based on aerogel-spun carbon nanotube yarn with a core-sheath structure. Compos. Part A Appl. Sci. Manuf..

[7] Lin Y.A., Zhao Y., Wang L., Park Y., Yeh Y.J., Chiang W.H., Loh K.J. (2020). Graphene K-Tape Meshes for Densely Distributed Human Motion Monitoring. Adv. Mater. Technol..

[8] Chowdhury A.R., Abdullah A.M., Hussain I., Lopez J., Cantu D., Gupta S.K., Mao Y., Danti S., Uddin M.J. (2019). Lithium doped zinc oxide based flexible piezoelectric-triboelectric hybrid nanogenerator. Nano Energy.

[9] Mahapatra B., Patel K.K., Vidya, Patel P.K. (2020). A review on recent advancement in materials for piezoelectric/triboelectric nanogenerators. Mater. Today Proc..

[10] Zang Y., Zhang F., Huang D., Gao X., an Di C., Zhu D. (2015). Flexible suspended gate organic thin-film transistors for ultra-sensitive pressure detection. Nat. Commun..

[11] Sun Q., Seung W., Kim B.J., Seo S., Kim S.W., Cho J.H. (2015). Active Matrix Electronic Skin Strain Sensor Based on Piezopotential-Powered Graphene Transistors. Adv. Mater..

[12] Xu M., Kang H., Guan L., Li H., Zhang M. (2017). Facile Fabrication of a Flexible LiNbO_3_ Piezoelectric Sensor through Hot Pressing for Biomechanical Monitoring. ACS Appl. Mater. Interfaces.

[13] Ning C., Dong K., Cheng R., Yi J., Ye C., Peng X., Sheng F., Jiang Y., Wang Z.L. (2020). Flexible and Stretchable Fiber-Shaped Triboelectric Nanogenerators for Biomechanical Monitoring and Human-Interactive Sensing. Adv. Funct. Mater..

[14] Choi D.Y., Kim M.H., Oh Y.S., Jung S.H., Jung J.H., Sung H.J., Lee H.W., Lee H.M. (2017). Highly Stretchable, Hysteresis-Free Ionic Liquid-Based Strain Sensor for Precise Human Motion Monitoring. ACS Appl. Mater. Interfaces.

[15] Souri H., Banerjee H., Jusufi A., Radacsi N., Stokes A.A., Park I., Sitti M., Amjadi M. (2020). Wearable and Stretchable Strain Sensors: Materials, Sensing Mechanisms, and Applications. Adv. Intell. Syst..

[16] Yin F., Ye D., Zhu C., Qiu L., Huang Y. (2017). Stretchable, Highly Durable Ternary Nanocomposite Strain Sensor for Structural Health Monitoring of Flexible Aircraft. Sensors.

[17] Nie M., Xia Y.H., Yang H.S. (2018). A flexible and highly sensitive graphene-based strain sensor for structural health monitoring. Clust. Comput..

[18] Zein A.E., Huppé C., Cochrane C. (2017). Development of a Flexible Strain Sensor Based on PEDOT:PSS for Thin Film Structures. Sensors.

[19] Lu Y., Liu Z., Yan H., Peng Q., Wang R., Barkey M.E., Jeon J.W., Wujcik E.K. (2019). Ultrastretchable Conductive Polymer Complex as a Strain Sensor with a Repeatable Autonomous Self-Healing Ability. ACS Appl. Mater. Interfaces.

[20] Huang L., Wang H., Wu P., Huang W., Gao W., Fang F., Cai N., Chen R., Zhu Z. (2020). Wearable Flexible Strain Sensor Based on Three-Dimensional Wavy Laser-Induced Graphene and Silicone Rubber. Sensors.

[21] Song P., Wang G., Zhang Y. (2021). Preparation and performance of graphene/carbon black silicone rubber composites used for highly sensitive and flexible strain sensors. Sens. Actuators A Phys..

[22] Yee M.J., Mubarak N., Abdullah E., Khalid M., Walvekar R., Karri R.R., Nizamuddin S., Numan A. (2019). Carbon nanomaterials based films for strain sensing application—A review. Nano-Struct. Nano-Objects.

[23] Lee J., Lim M., Yoon J., Kim M.S., Choi B., Kim D.M., Kim D.H., Park I., Choi S.J. (2017). Transparent, Flexible Strain Sensor Based on a Solution-Processed Carbon Nanotube Network. ACS Appl. Mater. Interfaces.

[24] Mehmood A., Mubarak N., Khalid M., Walvekar R., Abdullah E., Siddiqui M., Baloch H.A., Nizamuddin S., Mazari S. (2020). Graphene based nanomaterials for strain sensor application—A review. J. Environ. Chem. Eng..

[25] Xu J., Wang H., Ma T., Wu Y., Xue R., Cui H., Wu X., Wang Y., Huang X., Yao W. (2020). A graphite nanoplatelet-based highly sensitive flexible strain sensor. Carbon.

[26] Askari H., Asadi E., Saadatnia Z., Khajepour A., Khamesee M.B., Zu J. (2018). A flexible tube-based triboelectric–electromagnetic sensor for knee rehabilitation assessment. Sens. Actuators A Phys..

[27] Jha A.K., Lamecki A., Mrozowski M., Bozzi M. (2020). A Highly Sensitive Planar Microwave Sensor for Detecting Direction and Angle of Rotation. IEEE Trans. Microw. Theory Tech..

[28] Zhuo H., Hu Y., Tong X., Chen Z., Zhong L., Lai H., Liu L., Jing S., Liu Q., Liu C. (2018). A Supercompressible, Elastic, and Bendable Carbon Aerogel with Ultrasensitive Detection Limits for Compression Strain, Pressure, and Bending Angle. Adv. Mater..

[29] Lu S., Ma J., Ma K., Wang X., Wang S., Yang X., Tang H. (2019). Highly sensitive graphene platelets and multi-walled carbon nanotube-based flexible strain sensor for monitoring human joint bending. Appl. Phys. A.

[30] Huang J., Li D., Zhao M., Lv P., Lucia L., Wei Q. (2019). Highly stretchable and bio-based sensors for sensitive strain detection of angular displacements. Cellulose.

[31] Mu Y., Feng R., Gong Q., Liu Y., Jiang X., Hu Y. (2021). A Flexible Two-Sensor System for Temperature and Bending Angle Monitoring. Materials.

[32] Laflamme S., Saleem H.S., Vasan B.K., Geiger R.L., Chen D., Kessler M.R., Rajan K. (2013). Soft Elastomeric Capacitor Network for Strain Sensing Over Large Surfaces. IEEE/ASME Trans. Mechatron..

[33] Kharroub S., Laflamme S., Song C., Qiao D., Phares B., Li J. (2015). Smart sensing skin for detection and localization of fatigue cracks. Smart Mater. Struct..

[34] Kong X., Li J., Collins W., Bennett C., Laflamme S., Jo H. (2018). Sensing distortion-induced fatigue cracks in steel bridges with capacitive skin sensor arrays. Smart Mater. Struct..

[35] Liu H., Yan J., Kollosche M., Bentil S.A., Laflamme S. (2020). Surface Textures for Stretchable Capacitive Strain Sensors. Smart Mater. Struct..

[36] Liu H., Laflamme S., Li J., Bennett C., Collins W., Downey A., Ziehl P., Jo H. (2021). Investigation of Surface Textured Sensing Skin for Fatigue Crack Localization and Quantification. Smart Mater. Struct..

[37] Jeong J.H., Xu J., Jo H., Li J., Kong X., Collins W., Bennett C., Laflamme S. (2018). Development of wireless sensor node hardware for large-area capacitive strain monitoring. Smart Mater. Struct..

[38] ANSYS® (2020). Academic Research Mechanical, Release 19.1, Help System, Coupled Field Analysis Guide.

[39] Totten G.E. (2017). ASM Handbook Volume 18: Friction, Lubrication, and Wear Technology.

